
JOURNAL INFORMATION
==============================
NLM Title Abbreviation: Atlanta Med Surg J (1884)
NLM Journal ID: 101251623

Atlanta Medical and Surgical Journal (1884)

Publisher: Biomedical Journal Digitization Project

ARTICLE INFORMATION
==============================
PMCID: PMC9201839
Article version: 1
Subjects: News and Notes

News and Notes

Print publication date: 1899 Jan
Volume: 15
Volume Series: New Ser
Issue: 11
First page: 757
Last page: 765
License: This work is free of known copyright restrictions. For more information, please see PMC Back Issue Digitization.
License URL: https://creativecommons.org/publicdomain/mark/1.0/

==============================

### *NEWS AND NOTES.*

**NOTICE.—This JOURNAL and THE INTERNATIONAL JOURNAL OF SURGERY one year for $2.00 cash.**

IN Dr. Hutchins' article, page 658, line 10, read "*had no,*" for "*had.*" Page 661, line 6, "cicatrization," for "*scarification.*"

DR. T. H. HUZZA, of this city, died last month in the New York Hospital as a result of appendicitis. Dr. Huzza as a student at college made one of the most brilliant records that has ever been known. For a number of years he assisted Dr. Battey, in Rome, Ga., at his private infirmary.

DR. HENRY C. DOUGHTY died at his home in Augusta, Ga., last month at the early age of 26. It is supposed that Dr. Doughty's death was the result of tuberculosis, produced by the inhalation of these germs through his work at the college laboratory.

DR. L. W. PITCHFORD, of Estaboga, Ala., died on Sunday, November 27th, of typhoid fever. Dr. Pitchford was an alumnus of the old Southern Medical College.

THAT excellent journal, *The Louisville Journal of Medicine and Surgery,* has reduced its subscription price from two to one dollar a year. This seems to be the policy of a great number of medical journals for the coming year.

JUDGE MILLER, of Louisville, has handed down his decision in the injunction case of Drs. Moody & Kelly against Drs. Warthen,

Boyd and Cochrane, of the Kentucky School of Medicine. The Judge has decided that the election of the latter named gentlemen, Drs. Warthen, Boyd and Cochrane, to the positions claimed by them, was valid and therefore he dismissed the injunction. It is said that the case will be carried to the Court of Appeals.

---

***NOTICE.—This JOURNAL and THE INTERNATIONAL JOURNAL OF SURGERY one year for $2.00 cash.***

---

THE odor of onions may be removed by eating a sprig of parsley.

---

A SPRAINED ankle kept in hot water for twenty-four hours will heal.

---

A LEADING physician says that pepper is deadly poison to the system.

---

POWDERED chalk and orris root is one of the best dentifrices known.

---

AS MANY as 4,061 muscles have been counted in the body of a moth.

---

A STRONG solution of borax, applied twice a day, will cure ringworms.

---

AN authority says that the growth of children takes place entirely when they are asleep.

---

IT is a strange fact that injuries to the tongue, whether of man or animal, heal more quickly than those of any other part of the system.

GLYCERINE and lime juice is very good for preventing dandruff, and is nourishing to the hair.

---

A DROP of oil of cloves on a piece of cotton applied to the tooth twice a day will cure toothache.

---

A LITTLE spirits of camphor put in the water will prevent the face from looking greasy in hot weather.

---

MORE cases of consumption appear among needle-makers and file-makers than among any other class of laborers.

---

***NOTICE.—This JOURNAL and THE INTERNATIONAL JOURNAL OF SURGERY one year for $2.00 cash.***

---

THE Government has commemorated the sacrifice of the first American soldier on Cuban soil, by naming the general hospital attached to Camp Hamilton at Lexington, Ky., the John Blair Gibbs Hospital.

---

THE trustees of Columbia University have established in the College of Physicians and Surgeons a scholarship to be known as the O'Dwyer Scholarship, in recognition of Dr. Joseph O'Dwyer's professional labors.

---

PROFESSOR R. H. CRITTENDEN has been appointed to succeed Professor Geo. J. Brush, who has recently resigned his position as director of the Sheffield Scientific School, and accepted an appointment as Professor Emeritus.

---

THE Sternberg Hospital at Camp Thomas, Chickamauga Park, was closed November 14th. The twenty-eight patients in the hospital were sent to Fort Thomas, Ky., and the medical corps transferred to the hospital at Savannah.

---

FT. MONROE will soon be one of the most important military hospital stations in the country. The hospital is named the Josiah Simpson Hospital, and has during the past four months accommodated 1,500 sick and wounded soldiers, of whom about 500 still remain. It is now proposed to increase the capacity of the hospital to 2,500 beds by the erection of five additional buildings.

---

DR. MARY H. MURRAY has been appointed by the New York Board of Health medical inspector of the twelve public schools in the third ward of the borough of Queens, New York City. Dr. Murray is a graduate of the Woman's Medical College of the city of New York, and served as interne in the hospital and dispensary for one year. She is the first woman honored with an appointment on the staff of school inspectors.

---

PRESIDENT LOW of the Columbia University has issued an order approved in a meeting of the faculty of the College of Physicians and Surgeons, prohibiting smoking or spitting about the corridors or in the rooms of the buildings, except in the retiring-room and a designated part of the dissecting-room. The majority of men who smoke expectorate, and as this is now regarded not only as a filthy habit but as one which encourages the spread of disease, the new order will doubtless work a favorable reform in the habits of the medical students.

---

THE president of the board of managers of Craig Colony offers a prize of $100 for the best contribution to the pathology and treatment of epilepsy, originality being the main condition. The prize is open to universal competition, but all manuscripts must be submitted in English. All papers will be passed upon by a committee to consist of three members of the New York Neurological Society, and the award will be made at the annual meeting of the board of managers of Craig Colony, October 10, 1899. Manuscripts should be sent to Dr. Frederick Peterson, 4 West Fiftieth street, New York City, on or before September 1, 1899.

---

The announcement of the birth of a son to the Duchess of Aosta is declared to be another triumph for Dr. Schenck, the embryologist, who recently announced his discovery of the secret of sex, or the principles which regulate the production of male children. The Duchess of Aosta is said to have been a patient of Dr. Schenck, and to his careful treatment is due the birth of an heir to the Italian throne. The Duchess of Aosta is the third woman of high rank to demonstrate the efficacy of Dr. Schenck's treatment. The first was the Archduchess Frederick of Austria, and the second the Countess of Warwick. It is stated that Dr. Schenck has been in attendance upon a number of royal women who desire to bear sons for the inheritance of great titles.

---

GENERAL WOOD is indefatigable, in sun and rain alike. The result of his labors is apparent in every branch of the service—civil and military. The patients are reaping the result in better attendance and improved food and cooking. People who have lived here a lifetime are unable to understand the extraordinarily low death-rate, averaging for October, up to date, ten a day. During the same period of last year the deaths averaged 183 a day. The 300 prisoners in Santiago prison will in future be made to work on building roads for the city. The rock will be hauled over the railroad from the Songo quarries. It will be delivered at the rate of forty cents a ton, and the railroad officials have offered to deliver 500 tons of rock free as their share of the work of improving the city.—*New York World.*

---

SINCE 1872 the Mt. Sinai Hospital, New York, has occupied its present quarters at Sixty-sixth street and Lexington avenue. For more than ten years the present structure has proven inadequate to the demands made upon it, and a year ago the directors of the hospital decided to build a new and more modern structure. A site was purchased on Fifth avenue, embracing the entire square bounded by One Hundredth, One Hundred and First streets, Madison and Fifth avenues, with the exception of three city lots. Voluntary subscriptions are sought to defray the cost of the buildings, about $350,000 having been collected up to this time, we are informed. It is proposed to spend twice this sum for the building, which is to be equipped as a modern scientific hospital in the fullest sense of the term. Ground will shortly be broken, and it is expected that the new hospital will be occupied by 1900.

---

CRAMER (*Centralb. für Gynäkol.,* October 1, 1898) reports a case of fatal poisoning from the use of three pints of one per cent. solution of lysol as a uterine douche. Several cases of death following the injection of pure lysol have been reported, while in three instances its application upon skin wounds has resulted fatally, but so far as known this is the first fatal case from the use of a diluted solution of lysol. The uterine injection was made immediately after the birth of the placenta but was suddenly interrupted by the collapse of the patient. She recovered from the collapse, however, only to die ten days later with symptoms similar to those of carbolic-acid poisoning. Cramer reckoned that a quantity of lysol equal to seven minims of the drug may have found entrance to the venous circulation. The value of uterine injections depends not so much upon the disinfecting power of the fluid used as it does upon the mechanical cleansing. Hence, the importance of having the antiseptic fluid as weak as possible in case irrigation is employed after childbirth. Indeed, for this purpose, many prefer normal salt solution to any disinfectant.

---

THE lessons of some of our recent wars ought to be instructive to those who have the responsbility of placing a number of armed citizens in the field. Hitherto the lessons of war have been disregarded, though the teachings have been most emphatic. Disease kills more than the sword. This appears to be the inevitable course of wars. It was written in large letters in the Franco-Prussian war, and more fearfully still in the Crimean war. Since then in the Spanish-American war the ravages of yellow fever have been appalling, and now we learn that a telegram from Alexandria states that the men who have returned from the Nile continue to die like flies, from enteric fever, contracted possibly through the use of tinned beef, and certainly aggravated by immoderate indulgence in cheap, nasty spirits at fifteen-pence a bottle, the men having accumulated money in their pockets. The return states that every bed in the great hospital at Raseltin is occupied, besides which dozens of deaths occur weekly, and it is believed that ten per hundred of the British troops on the Nile are already affected. The disease is, moreover, following the troops into Crete. When tinned beef is fresh it is all right, but big tins rapidly spoil before the contents are consumed. The mortality during the whole campaign is probably less than the subsequent' deaths from enteric fever.—*The Scalpel.*

---

ONE of the gunners of Admiral Schley's fleet is said to have lost his reason as a result of the naval battle of July 3, with Cervera's squadron. He is now in St. Elizabeth's Insane Asylum, Washington, D. C., undergoing treatment. The patient's trouble began with concussion of the brain, caused by the detonation of the heavy guns fired during the fight. This is regarded as an unusual case. The report set forth that eight of the ten casualties upon the American ships in the battle of Santiago were ruptures of the ear drums, caused by the terrific cannonading. One of the cases was that of Lieutenant Harrison of the Oregon, who stuck his head out of a turret just as a thirteen-inch rifle was fired. The noise and shock of the explosion lacerated the drum of both ears, and for a time he was totally deaf. Under the careful treatment of the naval surgeons he is gradually recovering his hearing and will in all probability be as well as ever in a few more months. All the other men who were similarly affected have been cured and have returned to their ships for duty. During the fight at Manila there is no record of any of Admiral Dewey's men receiving injuries of this sort. The explanation of this is simple. The gunners on battleships are accustomed to plug their ears with cotton to protect them before firing begins, but at Santiago there was no chance for precautions of this kind. The men were on deck for inspection at the time the enemy was discovered issuing from the harbor, and they simply rushed to their guns and began blazing away without thought of cotton or anything else. At Manila the men were all prepared for the fight, every precaution having been taken to prevent injury to ear drums, etc.

---

The Southern Medical College Association met at Memphis, Tenn., December 5, prior to the meeting of the Southern Surgical and Gynecological Association, and after considerable discussion, it was decided to increase the course of study from three to four years. It is well known that the question of making the course four years instead of three has been before the association for two years, but it has been passed from time to time, in order that so radical a move might be fully discussed by all the faculties that are to be affected. Dr. Sinclair of Memphis presented the matter first at the meeting of the association in Atlanta, and since then from time to time it has been discussed frequently among each of the faculties in the association. At the meeting, only two of the thirteen colleges in the association failed to send representatives to the conference. On account of the change to the four-year course, it was necessary to introduce a number of amendments to the constitution and by-laws of the association, but none of them was passed, because it is the rule that such amendments shall lie over for one year. This is for the purpose of giving the delegates an opportunity to present plans for whatever changes are contemplated in their respective faculties before final action is taken. The delegates present were: Drs. S. S. Crockett, of the University of Nashville; W. E. B. Davis, of the Birmingham Medical College; Christopher Thompkins, of the Virginia Medical College at Richmond; Ernest S. Lewis, of Tulane University, New Orleans; G. C. Savage, and Richard Douglas, of the Medical Department of Vanderbilt University of Nashville; T. H. Wood, of the University of Tennessee, at Nashville; Campbell of the Tennessee Medical College, at Knoxville; G. A. Ketchum, of the Alabama Medical College of Mobile, and A. G. Sinclair, of the Memphis Medical College. The University of the South was to have been represented by Dr. J. S. Cain of Nashville, but he sent a proxy in the person of Dr. Savage. The only two colleges in the association not represented were the Texas Medical College of Galveston and the Southern Medical College of Atlanta. The other absentee from the list of thirteen was the Bessemer Medical College of Bessemer, Ala., and it is no longer in existence.—*Journal of A. M. A.*
